# Statistical morphological analysis reveals characteristic paraspinal muscle asymmetry in unilateral lumbar disc herniation

**DOI:** 10.1038/s41598-021-95149-6

**Published:** 2021-08-02

**Authors:** Yiming Xiao, Maryse Fortin, Joshua Ahn, Hassan Rivaz, Terry M. Peters, Michele C. Battié

**Affiliations:** 1 Department of Computer Science and Software Engineering, Concordia University, Montreal, Canada; 2 PERFORM Centre, Concordia University, Montreal, Canada; 3 Health, Kinesiology and Applied Physiology, Concordia University, Montreal, Canada; 4 Department of Kinesiology, Western University, London, Canada; 5 Department of Electrical and Computer Engineering, Concordia University, Montreal, Canada; 6 Robarts Research Institute, Western University, London, Canada; 7 School of Physical Therapy and Western’s Bone and Joint Institute, Western University, London, Canada

**Keywords:** Musculoskeletal system, Neuromuscular disease, Biomarkers, Biomedical engineering, Mathematics and computing

## Abstract

Growing evidence suggests an association of lumbar paraspinal muscle morphology with low back pain (LBP) and lumbar pathologies. Unilateral spinal disorders provide unique models to study this association, with implications for diagnosis, prognosis, and management. Statistical shape analysis is a technique that can identify signature shape variations related to phenotypes but has never been employed in studying paraspinal muscle morphology. We present the first investigation using this technique to reveal disease-related paraspinal muscle asymmetry, using MRIs of patients with a single posterolateral disc herniation at the L5-S1 spinal level and unilateral leg pain. Statistical shape analysis was conducted to reveal disease- and phenotype-related morphological variations in the multifidus and erector spinae muscles at the level of herniation and the one below. With the analysis, shape variations associated with disc herniation were identified in the multifidus on the painful side at the level below the pathology while no pathology-related asymmetry in cross-sectional area (CSA) and fatty infiltration was found in either muscle. The results demonstrate higher sensitivity and spatial specificity for the technique than typical CSA and fatty infiltration measures. Statistical shape analysis holds promise in studying paraspinal muscle morphology to improve our understanding of LBP and various lumbar pathologies.

## Introduction

Low back pain (LBP) is among the most common musculoskeletal problems in adults, yet the underlying source of pain most often remains unknown, hindering effective prevention and treatment efforts^1^. To improve the understanding of painful spinal disorders and their prognoses, as well as developing appropriate treatment strategies, there is a growing interest in their association with lumbar paraspinal muscle morphometry and composition, using magnetic resonance imaging (MRI). Commonly viewed as an indicator for muscle force production capacity, muscle cross-sectional area (CSA) is often used as a key measure to assess paraspinal muscle status, as is bilateral paraspinal muscle asymmetry. Paraspinal muscle composition (e.g., fatty infiltration) is also commonly used as an indicator of muscle health. In many existing case–control studies^2,3^ that compare patients with healthy controls, LBP and painful lumbar pathologies have been associated with smaller muscle size and/or greater fatty infiltration^4^. However, previous findings have not been consistent, which is likely at least in part due to variations in muscle measurement methodology across studies and differences in clinical presentation, as well as variable adjustment of possible confounding factors, such as age and sex^5^.

Asymmetry in paraspinal muscles has been linked with unilateral LBP and lumbar disorders, which provide a model to gain insights into the etiology and pathology of these disorders. Studies^6–10^ that examine disease-related muscle asymmetry have primarily focused on the multifidus muscle. Previously, with a porcine model, Hodges et al.^11^ demonstrated rapid level- and side-specific multifidus muscle atrophy and adipocyte enlargement resulting from anterolateral disc or nerve root lesions. In their study of multifidus asymmetry, Battie et al.^7^ found greater total multifidus CSA ipsilateral to the pathology at the level of disc herniation, and greater fat infiltration on the side of disc herniation both at and below the level of pathology. Later, Fortin et al*.*^8^ investigated the asymmetry of the multifidus and erector spinae for posterolateral disc herniation at L4-L5. Although no significant CSA asymmetry of the multifidus was found at spinal levels above, at, or below the disc herniation, greater fat infiltration was reported on the side of the pathology at the adjacent level. To date, reports^6,9^ regarding multifidus asymmetry have not been consistent, particularly for muscle size and location of findings with respect to the level of pathology. If multifidus asymmetry is side- and level-specific in patients with symptomatic disc herniations, characteristic muscle morphology (i.e., shape) may serve as a helpful biomarker to localize less obvious disc pathologies responsible for disc-related symptoms.

Although quantitative measurements of muscle CSA are commonly used to examine their relationship with different lumbar pathologies that contribute to pain, these metrics fail to describe local variations in morphometric properties of the muscle groups beyond their sizes across targeted populations. Statistical shape models (SSMs)^12^ provide a compact description for the shapes of a collection of similar objects, by using data dimension reduction with principal component analysis (PCA). The technique allows extraction of uncorrelated shape variations (modes) from the population under study and quantifies their contributions to the total variation from the mean shape. Compared with CSA measures, this technique has two major advantages. First, it can identify detailed local and global geometric variations in a low-dimensional representation, which can be used to reveal phenotype-specific muscle shape characteristics. Second, the technique allows visualization of individual shape variations, which offer intuitive assessment of the morphometric properties. This technique has been successfully employed to study phenotype-related shape variations and alterations in various organs of the human body, including the heart^13^, brain^14,15^, and bones^16^, providing valuable biomarkers for diagnosis and prognosis purposes. More recently, Deane et al.^17^ investigated the link between intrinsic lumbar spinal bone shape and lumbar disc degeneration using this technique. Their study provides new evidence that intrinsic spinal shape phenotypes are associated with lumbar disc degeneration and quality of life in patients. However, to the best of our knowledge, statistical shape models have not been employed to study morphometric characteristics of lumbar paraspinal muscles in relation to pathology.

In this study, we present the first exploratory investigation using statistical shape analysis to reveal asymmetry of paraspinal muscles in a cohort of patients with single unilateral disc herniation at the L5-S1 level. More specifically, statistical shape models were constructed for the multifidus and erector spinae muscles segmented from axial MRI slices at the L5-S1 and S1 levels, and resulting shape variations were correlated with phenotypes, including disease status, sex and age. As a comparison, conventional measures of bilateral CSAs and level of fat infiltration were analyzed. We hypothesized that unilateral disc herniation is associated with asymmetry in paraspinal muscle shapes, and that statistical shape models would provide a more sensitive and detailed characterization of muscle morphometry in analyzing disease-related variations.

## Methods and materials

### Patient samples

In total, 24 patients (11 females and 13 males, age = 41.5 ± 7.9 years old) were retrospectively selected from the European research consortium project, Genodisc, on commonly diagnosed lumbar pathologies (physiol.ox.ac.uk/genodisc). Included subjects had a single level posterolateral disc herniation at L5-S1 with concomitant leg pain suggestive of radiculopathy on the side of the herniation. No other spinal conditions were noted and no patients received prior surgical treatments for disc herniation at the time of imaging. The herniation and leg pain were on the left in 15 of the 24 cases and the durations of leg pain and back pain were 10.7 ± 14.4 months and 14.2 ± 25.1 months, respectively. T2-weighted (T2w) MRI scans of the lumbar muscles were used for all patients, and axial slices of the L5-S1 and S1 levels were acquired. All MRI data were obtained with the patients in the supine position. The MRI was cropped at the S1 level for two subjects and due to the limited field of view, we obtained only 22 MRI slices at S1 for analysis, rather than the full 24 MRI slices as available for L5-S1. All patients provided informed consent, acknowledging that their data would be used for research to better understand and characterize common spinal disorders. The study was approved by the Ethics Research Board of Western University. The primary Genodisc data were obtained during the ECFP7 Project (HEALTH-F2-2008-201626). All methods were performed in accordance with the relevant guidelines and regulations.

### Image preprocessing and segmentation

**Figure 1:**
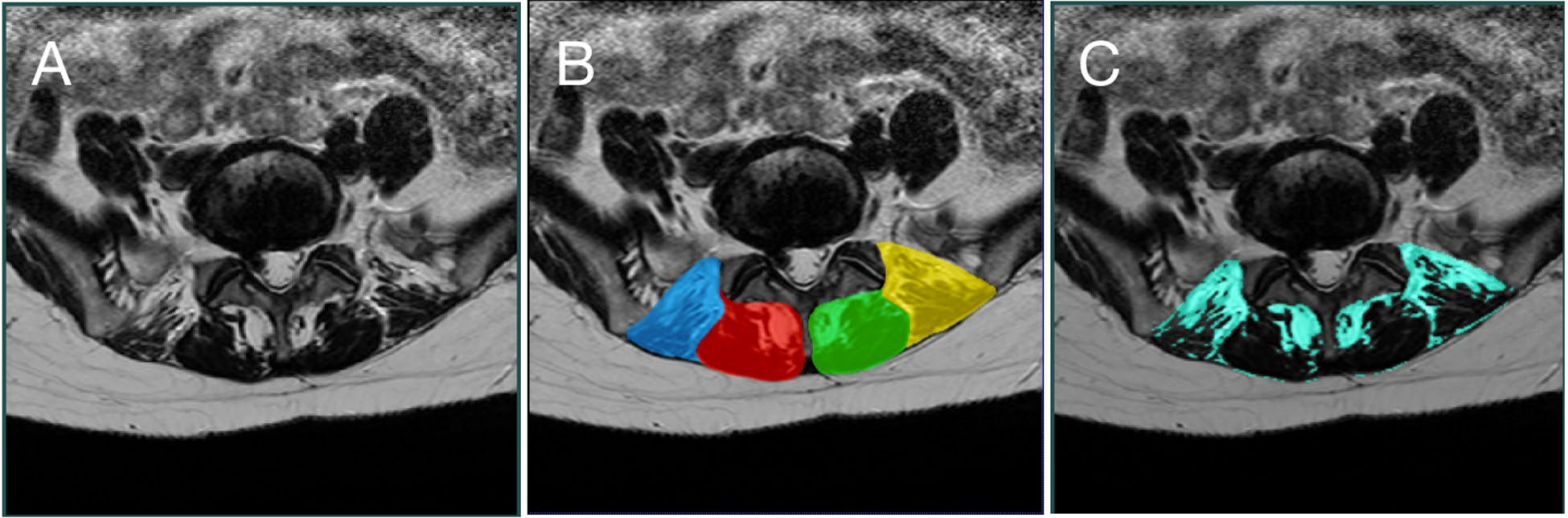
Demonstration of paraspinal muscle and fat tissue segmentation at the L5-S1 level. (**A**) Processed MRI, (**B**) manual segmentation of paraspinal muscles; (**C**) automatic fat tissue segmentation.

All axial cross-sectional images were corrected for MRI intensity anomalies due to radiofrequency field inhomogeneities in the scanner^18^. Then image intensity standardization was performed^19^ so that each scan exhibited a unified image contrast and intensity range. For each cross-sectional MRI, multifidus and erector spinae muscles were manually segmented bilaterally using the medical image segmentation software, ITK-SNAP (*itksnap.org*). Segmentation was conducted by JA, who was familiar with the relevant anatomy and had segmented over 300 cross-sectional MRIs of paraspinal muscles at different levels. A representation of the segmentation is shown in Fig. 1. To further verify the segmentation quality, 10 patients were randomly selected from the cohort. Their MRIs at the L5-S1 and S1 levels were re-segmented by JA and YX to assess inter- and intra-rater variability using Dice coefficient, which ranges 0 ~ 1, with 1 indicating perfect agreement between two segmentations and 0.85 meaning excellent overlap. Furthermore, for intra-rater variability evaluation, a minimum of 48 h were ensured between two sets of segmentations by JA. The complete results of intra- and inter-rater segmentation variability are detailed in Table S1 in the *Supplement Information*. Overall, the intra- and inter-rater variability was measured above 0.90 on average for all muscle groups at both L5-S1 and S1 levels, indicating excellent manual segmentation quality.

### Muscle size and composition measurement

Cross-sectional areas were computed using manual segmentation of the left and right multifidus and erector spinae muscles. By using manually segmented muscle CSA to constrain the regions of interest, fatty cross-sectional areas were segmented with the k-means algorithm^20^. The algorithm can automatically separate fat and lean muscle based on their intensity difference. Visual quality control for the results was performed by the author YX, and all results were deemed of good quality. Finally, fat % relative to CSA was computed for multifidus and erector spinae for all patients at the L5-S1 and S1 levels. A demonstration of the fat area segmentation is shown in Fig. 1C.

### Statistical shape modelling

**Figure 2:**
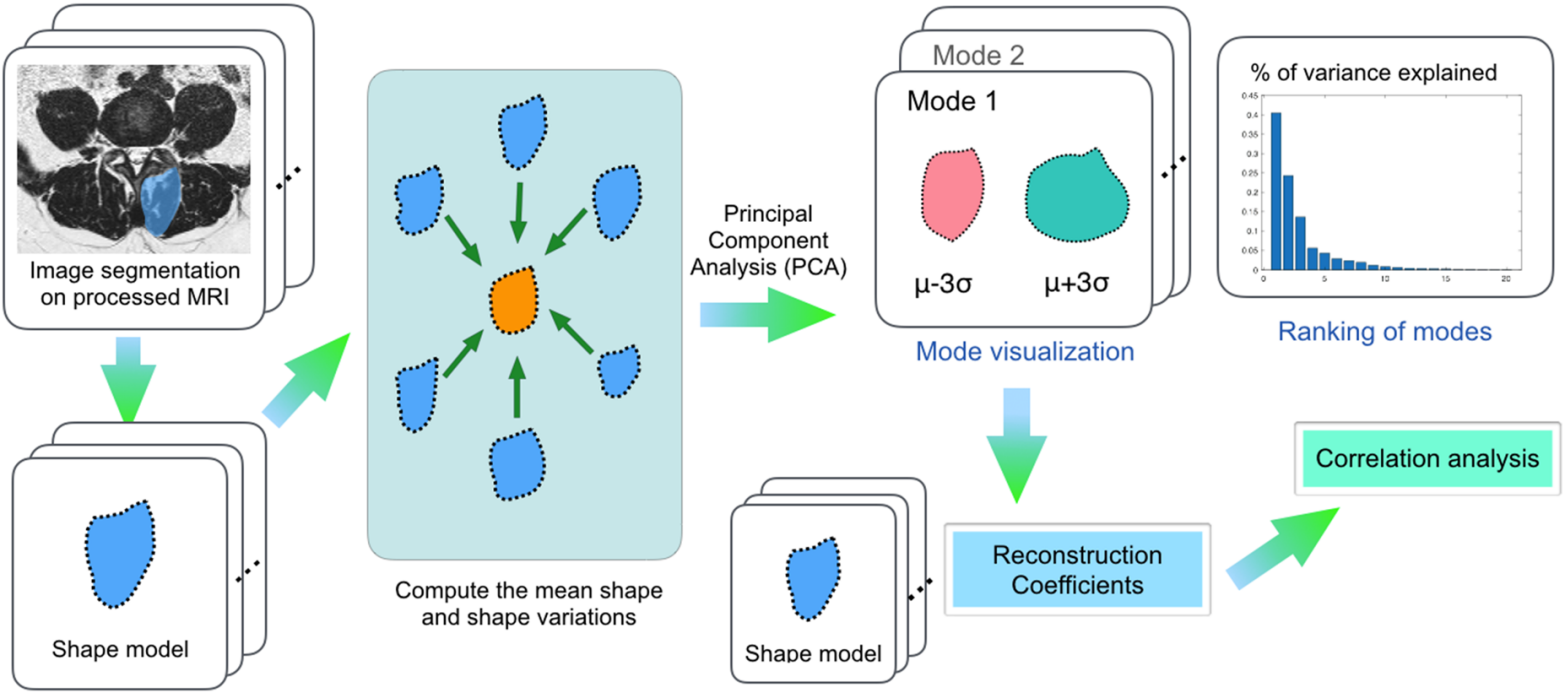
Demonstration of statistical shape analysis for paraspinal muscles.

Statistical shape models^12^ can be used to characterize the morphological property of a muscle group among a target population. For this study, we built the SSMs for the multifidus, as well as for the erector spinae at the L5-S1 and S1 levels. They model intrinsic anatomical variations across the population and shape alterations that were correlated with disc herniation. The procedure of statistical shape modelling is demonstrated in Fig. 2. To establish the common coordinates needed for shape analysis and to help visualize the results, population-averaged symmetric MRI templates were constructed for each spinal level by deforming and averaging all relevant MR images and their left–right mirrored versions using group-wise nonlinear registration^21^. To normalize the global individual size differences and reveal localized shape variations, the muscle segmentations (original and mirrored versions) were linearly registered to those of the respective MRI templates. With statistical shape analysis of the aligned muscle segmentations, the dominant principal shape variations (or modes) explaining 90% of the morphometric variations were extracted for further analysis. More specifically, the reconstruction coefficients for the dominant shape modes, which represent the magnitude of contribution from the modes to an individual shape, were obtained for all subjects with respect to the multifidus and erector spinae at both levels. The resulting coefficients were then correlated with the clinical and demographic factors to further identify associated trends in morphological variations.

### Statistical analysis

To investigate the commonly used measures of muscle morphology and composition, Spearman partial correlation was computed to examine CSAs and fat infiltration (i.e., fat %) of the multifidus and erector spinae between the affected and unaffected sides at the L5-S1 and S1 levels for the entire cohort, while controlling for the effects of sex and age, which are known to affect paraspinal muscle morphometry. To verify the intrinsic impact from sex and age in the cohort, CSAs and muscle composition from both affected and unaffected sides were correlated with one of the factors while controlling for the effects of the other and the disease influence. For all tests, correlations with a *p*-value < 0.05 were considered statistically significant.

**Figure 3:**
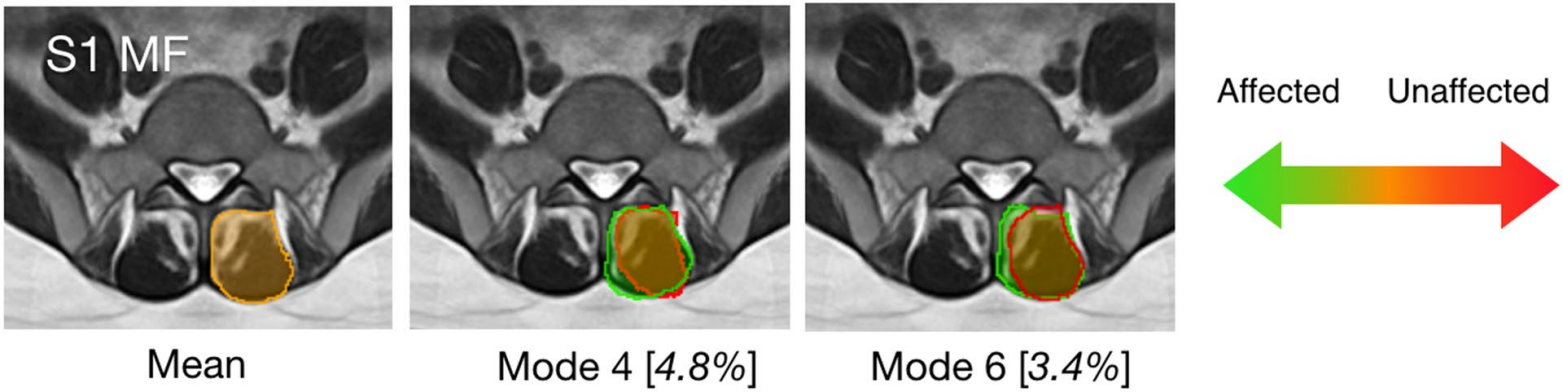
Modes of the multifidus (MF) shape that were associated with the disc herniation at S1. The mean shape is shown in orange overlaid on the population-averaged MRI template. The trend of shape variation towards the muscle being affected is in green and the opposite is in red. For each mode, threefold standard deviations from the mean shape are depicted for both directions of shape variants, and the percentages of shape variance explained are included in [] for all modes.

**Figure 4:**
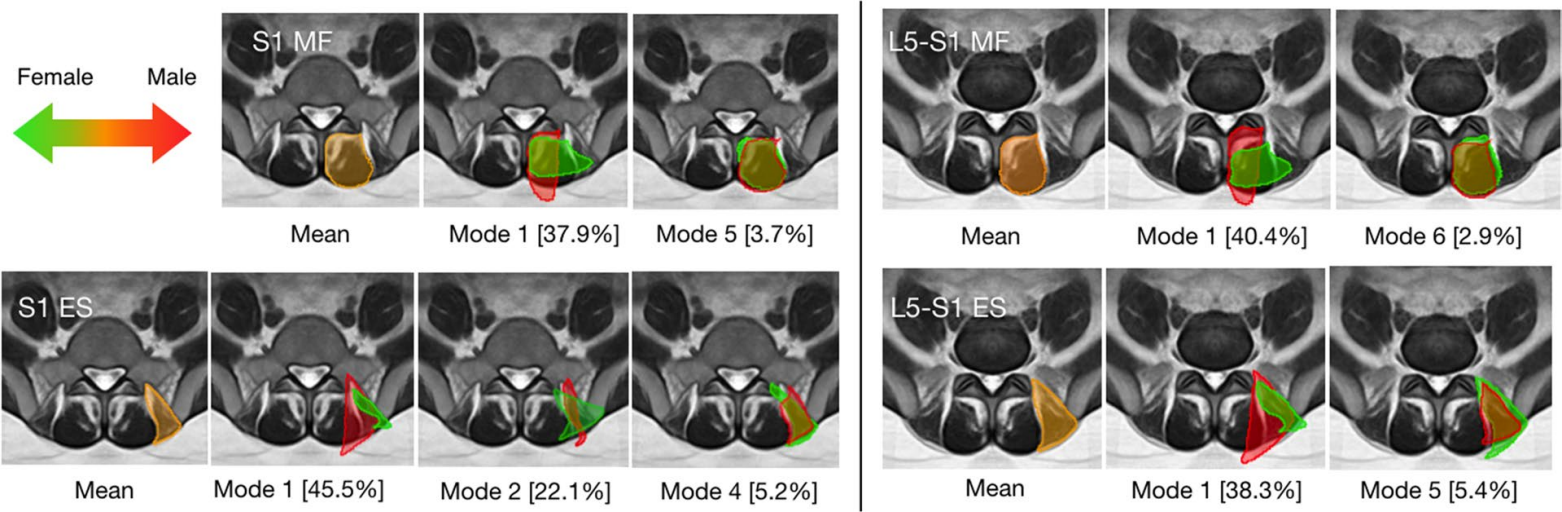
Modes of the multifidus (MF) and erector spinae (ES) shape associated with sex. The mean shape is shown in orange overlaid on population-averaged MRI templates. The trend of shape variation towards female sex is in green and the opposite is in red. For each mode, threefold standard deviations from the mean shape are depicted for both directions of shape variants, and the percentages of shape variance explained are included in [ ] for all modes.

**Figure 5:**
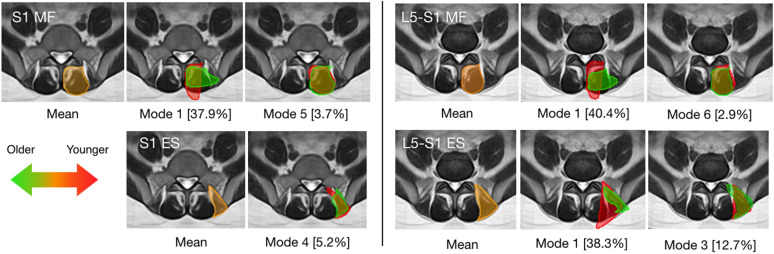
Modes of the multifidus (MF) and erector spinae (ES) shape that were associated with age. The mean shape is shown in orange overlaid on population-averaged MRI templates. The trend of shape variation towards an older age is in green and the opposite is in red. For each mode, threefold standard deviations from the mean shape are depicted for both directions of shape variants, and the percentages of shape variance explained are included in [ ] for all modes.

As for statistical shape analysis, Spearman partial correlation was performed to compare the reconstruction coefficients for the muscle shapes between the affected and unaffected side. For each test, the effects of age and sex were set as covariates and controlled in the analysis. The modes with significant correlations (p-value < 0.05) were identified, and their visual representations (Figs. 3, 4 and 5) were inspected to interpret the trends related to the pathology. Furthermore, anatomical variations due to sex and age were also examined in a similar manner. When testing each factor, the other demographic information and the factor of affected vs. unaffected side were set as covariates.

## Results

### Muscle size and composition measurement

The measured muscle CSAs and fat % on the affected and unaffected sides at different spinal levels are reported in Table 1. From the partial correlation results, no significant differences were found between the two sides. With regard to demographic factors, lower mean CSA and higher mean fat% in multifidus and erector spinae were found in female patients (see Table 2). However, within this limited sample, significant sex-related differences were found only in fat % of the multifidus at the L5-S1 and S1 levels and the erector spinae at L5-S1. In addition, older age was associated with less muscle CSA (*p* = *0.041*) and greater fatty infiltration (*p* = 0.022) in erector spinae at L5-S1, as well as higher erector spinae fat% (*p* = 0.0093) at S1.

**Table 1:** Measurements (mean ± sd) of CSA and fatty infiltration (Fat%) of the multifidus (MF) and erector spinae (ES) on the affected and unaffected sides.

	CSA (cm^2^) affected	CSA (cm^2^) unaffected	CSA p-value*	Fat% affected	Fat% unaffected	Fat% p-value*
L5-S1-MF	12.9 ± 2.0	12.8 ± 1.9	0.84	23.5 ± 6.0%	24.6 ± 7.5%	0.47
L5-S1-ES	10.0 ± 3.6	9.7 ± 2.8	0.76	38.4 ± 10.8%	39.0 ± 10.5%	0.91
S1-MF	13.0 ± 2.2	12.5 ± 1.8	0.41	27.1 ± 7.2%	27.0 ± 7.6%	0.82
S1-ES	6.6 ± 2.6	6.2 ± 2.2	0.67	41.7 ± 11.7%	40.6 ± 9.8%	0.92

*The *p*-values were obtained with Spearman partial correlation while controlling for the effects of sex and age.

**Table 2:** Measurements (mean ± sd) of CSA and fatty infiltration (Fat%) of the multifidus (MF) and erector spinae (ES) with respect to sex.

	CSA (cm^2^) male	CSA (cm^2^) female	CSA p-value*	Fat% male	Fat% female	Fat% p-value*
L5-S1-MF	13.0 ± 1.9	12.7 ± 1.9	0.75	20.8 ± 6.7%	27.8 ± 4.6%	0.00037^#^
L5-S1-ES	10.4 ± 2.6	9.3 ± 3.8	0.55	35.1 ± 8.8%	42.9 ± 11.0%	0.021^#^
S1-MF	13.0 ± 2.1	12.4 ± 1.8	0.86	24.2 ± 7.9%	30.4 ± 5.0%	0.011^#^
S1-ES	6.6 ± 1.8	6.2 ± 3.0	0.41	39.0 ± 10.6%	43.7 ± 10.4%	0.41

*The *p*-values were obtained with Spearman partial correlation while controlling for the effects of age and the side of pathology. Significant *p*-values are marked with “#”.

### Statistical shape analysis

The SSMs contain 6 modes that account for ~ 90% of the shape variations for the multifidus and erector spinae at the L5-S1 and S1 levels. For each SSM, the respective modes are ranked in a descending order of their contributions to the total shape variability (i.e., Mode 1 explains the highest percentage of variance). The top 6 modes for each muscle group at each level are demonstrated in Fig. S1 of the *Supplementary Information*. With statistical shape analysis, the relevant modes that are associated with demographic and clinical factors are listed in Table 3, and the full results for the analysis are listed in Table S2 of the *Supplementary Information*. Note that as SSMs offer statistically independent shape variations and partial correlation can remove the effects of covariates, each correlation in Table S2 represents an independent investigation. Thus, for our exploratory study, multiple comparison correction was not used, like previous statistical shape analysis studies^13,16,17^. However, we acknowledge that some of the discovered correlations with statistical significance may be subject to chance, and we will further validate these findings with more subjects in our future confirmatory studies. More specifically, disc herniation at L5-S1 was associated with Mode 4 (*p* = 0.032) and Mode 6 (*p* = 9.2 × 10^–3^) from the SSM of the multifidus at S1. These two modes are illustrated in Fig. 3 and accounted for 4.8% and 3.4% of the total shape variance of the multifidus at S1, respectively. Note that as both the original and mirrored MRI slices were used, we only performed the analysis on one side of the image. With the visualization, we can see that the multifidus on the affected side has additional area at the medial site near the spinous process. When correlated with the factors of sex and age, more associated modes (p < 0.05) were identified and demonstrated in Figs. 4 and 5. Overall, the modes that were associated with sex and age account for a higher proportion of variance explained, and a number of these modes are linked to both factors. For example, in Mode 1 of the multifidus at both L5-S1 and S1, a muscle shape elongated in the mediolateral direction correlated with the female sex and older age, while slimmer erector spinae (Mode 1) were associated with male sex and younger age. However, there was no overlap between the modes associated with disc herniation and demographic factors (i.e., sex and age).

**Table 3:** Significant modes (shape variations) of each muscle group with respect to different factors (*p* < 0.05).

	Affected vs. unaffected	Sex	Age
L5-S1-MF	No significant modes	Mode 1(*p* = 0.029), Mode 6 (*p* = 3.0 × 10^–4^)	Mode 1 (*p* = 2.0 × 10^–4^), Mode 6 (*p* = 2.0 × 10^–4^),
L5-S1-ES	No significant modes	Mode 1 (*p* = 0.025), Mode 5 (*p* = 2.3 × 10^–3^)	Mode 1 (*p* = 2.3 × 10^–3^), Mode 3 (*p* = 0.037)
S1-MF	Mode 4 (*p* = 0.032) Mode 6 (*p* = 9.2 × 10^–3^)	Mode 1 (*p* = 0.024), Mode 5 *(p* = 2.0 × 10^–4^),	Mode 1 (*p* = 0.030), Mode 5 (*p* = 0.045)
S1-ES	No significant modes	Mode 1 (*p* = 0.019), Mode 2 (*p* = 0.030), Mode 4 (*p* = 3.0 × 10^–5^)	Mode 4 (*p* = 0.033)

## Discussion

In our sample of 24 patients with disc herniation and longstanding associated radiating leg pain, neither CSAs nor fatty infiltration showed significant dependence on the side of the disc herniation. So far, existing studies^10^ on side-to-side differences of CSA in disc herniation have provided conflicting conclusions. Some^10^ reported smaller or larger CSA of the multifidus on the painful side, while others found no significant asymmetry^4^. Meanwhile, increased fatty infiltration in lumbar multifidus on the side of pathology has been demonstrated with relatively higher consistency in the literature^10^. Overall, we found no significant CSA asymmetry at L5-S1 and S1 or side-to-side differences in fatty infiltration. With respect to the effects of sex and age, previous studies^22^ have concluded that female sex and older age are associated with smaller CSA and higher fatty infiltration in lumbar paraspinal muscles. Our results are in agreement with these findings, regardless of the influence from disc herniation. In addition, at the S1 level, the ilium can constrain the geometries of the paraspinal muscles, and its shape and size can be influenced by sex^23^. Therefore, the geometry of the ilium can be a major contributor to the observed CSA and morphological differences (as revealed by statistical shape analysis) with respect to sex at the S1 level.

In contrast to the commonly employed paraspinal measures of CSA and fatty infiltration, where no significant results were found, statistical shape analysis identified significant shape variations in multifidus on the affected side. Among the dominant modes of the multifidus at S1, those that correspond to the influence of pathology account for 8.2% of the total variance. However, the modes corresponding to sex and age were responsible for higher contributions to the overall shape variance. The substantial influence of sex and age on muscle morphology may partially mask pathology-related CSA asymmetry in our study, and future investigations that place additional constraints in demographic factors (e.g., only male subjects) during cohort selection may be beneficial^24^.

As fatty infiltration may be indirectly associated with muscle morphology, an additional experiment was conducted to correlate fat% and the identified modes in Figs. 3, 4 and 5. While muscle fatty infiltration was not associated with any detected shape variations related to disc herniation (p > 0.05), significant correlations were revealed for certain modes related to demographic factors. More specifically, fat % was associated with Mode 1 of multifidus at L5-S1 and both muscle groups at L5-S1 and S1, where a more elongated multifidus and smaller (e.g., slimmer) erector spinae were linked to higher fatty infiltration in the corresponding muscle group. Furthermore, greater lateral area of multifidus at L5-S1 (Mode 6) was also positively correlated with higher fat%. Compared to CSA, statistical shape analysis allows more intuitive visualization of local morphological alterations that are subject to different factors, easier interpretation for the contribution of related shape variations, and potentially superior sensitivity in detecting pathology-related changes.

Although different descriptions for lumbar multifidus morphology exist^25^, Macintosh et al.^26^ found that the principal fascicles of the lumbar multifidus are innervated by a single nerve root that stretches 3 ~ 4 vertebral levels caudally, implying that disease-related changes should be mainly detected at the levels below and on the side of the pathology. This was demonstrated in several studies^11,24,27^ that investigated muscle asymmetry related to disc herniation and injuries. From statistical shape analysis, we observed side- and level-specific asymmetry that was correlated with symptomatic disc herniation at L5-S1. More specifically, the identified variations at the level below the pathology (i.e., S1) support previous reports^11,24,27^ and offer insights on the trend of local shape variations in addition to prior findings related to cross-sectional areas.

One major limitation of the presented study is that the included patient sample was relatively small. Therefore, we exclusively focused on one pathology at a single level. However, despite the limited sample, the exploratory investigation introducing statistical shape analysis to identify muscle morphological properties in lumbar pathology did reveal significant trends in multifidus shape asymmetry related to disc herniation. In the future, validation of this technique in paraspinal muscle morphometry with respect to larger patient populations, various lumbar pathologies, and additional spinal levels of pathology will be important. For this, automatic image segmentation algorithms based on deep learning techniques^20^ will be highly instrumental. Furthermore, intrinsic side-to-side anatomical differences may exist beyond the effects of sex and age and contribute to the identified shape asymmetry. A previous study^28^ reported that paraspinal muscle asymmetry greater than 10% is common in men without a history of LBP. Furthermore, behavioral, environmental, and genetic factors may also modulate muscle asymmetry^24^. These can further complicate the investigation, but in-depth analyses that de-couple these factors, as well as comparison of paraspinal muscle shapes between patients and healthy controls, are out of the scope of the present study. Future work will incorporate healthy participants in statistical shape modelling, as well as additional variates to further elucidate the effects of various factors on paraspinal muscle asymmetry, as well as the underlying interaction between lumbar pathologies, external factors, and muscle shapes.

In conclusion, we present the first investigation using statistical shape analysis to study the morphology of paraspinal muscles. Using a patient cohort with symptomatic unilateral L5-S1 disc herniation, we identified statistically significant and specific morphological asymmetry in the multifidus at the S1 level. With potentially superior sensitivity and spatial specificity, statistical shape analysis has demonstrated promise beyond simple CSA measurements in studying paraspinal muscle morphology as possible diagnostic and prognostic biomarkers, to improve our understanding of LBP and various lumbar pathologies.

## Supplementary Information

Supplementary Information.

## Data Availability

The datasets generated during and/or analyzed during the current study are available from the corresponding author on reasonable request.
