# Supplementary material for: Statistical morphological analysis reveals characteristic paraspinal muscle asymmetry in unilateral lumbar disc herniation
Source: Sci Rep. 2021 Aug 2;11:15576. doi: 10.1038/s41598-021-95149-6 (PMC8329062; doi:10.1038/s41598-021-95149-6)
Supplement: Supplementary file 1 — Supplementary Information. [file 41598_2021_95149_MOESM1_ESM.pdf]

Supplementary Information

Statistical morphological analysis reveals characteristic paraspinal muscle asymmetry in unilateral lumbar disc herniation

*Yiming Xiao[1,2], Maryse Fortin[2,3], Joshua Ahn[4], Hassan Rivaz[2,5], Terry M. Peters[6], and Michele C. Battié[7]

[1] Department of Computer Science and Software Engineering, Concordia University, Montreal, Canada
[2] PERFORM Centre, Concordia University, Montreal, Canada
[3] Health, Kinesiology and Applied Physiology, Concordia University, Montreal, Canada
[4] Department of Kinesiology, Western University, London, Canada
[5] Electrical and Computer Engineering, Concordia University, Montreal, Canada
[6] Robarts Research Institute, Western University, London, Canada
[7] School of Physical Therapy and Western's Bone and Joint Institute, Western University, London, Canada

*Corresponding author: Yiming Xiao (yiming.xiao@concordia.ca)

1. Intra-rater and inter-rater variability evaluation

Ten patients were randomly selected to assess intra- and inter-rater variability in muscle segmentation using Dice coefficient. In addition to the labels used in the study, the 10 patients' images were manually segmented by JA and YX for the multifidus and erector spinae. The inter-rater variability was computed from JA's two sets of segmentation while inter-rater variability was computed from JA's segmentation used in the study and that of YX. The results are in Table S1.

	Left MF	Right MF	Left ES	Right ES
L5-S1 Intra-rater	0.97±0.01	0.97±0.02	0.96±0.02	0.96±0.02
S1 Intra-rater	0.97±0.02	0.96±0.04	0.96±0.02	0.94±0.03
L5-S1 Inter-rater	0.95±0.01	0.95±0.01	0.94±0.02	0.94±0.03
S1 Inter-rater	0.96±0.02	0.96±0.02	0.92±0.04	0.92±0.04

Table S1. Intra- and inter-rater variability measured using Dice coefficient for the multifidus and erector spinae muscles at the L5-S1 and S1 levels.

2. Statistical shape modeling of paraspinal muscles

The top 6 modes (shape variations) that account for ~90% of the variance for each muscle group at each level are shown in Fig. S1.

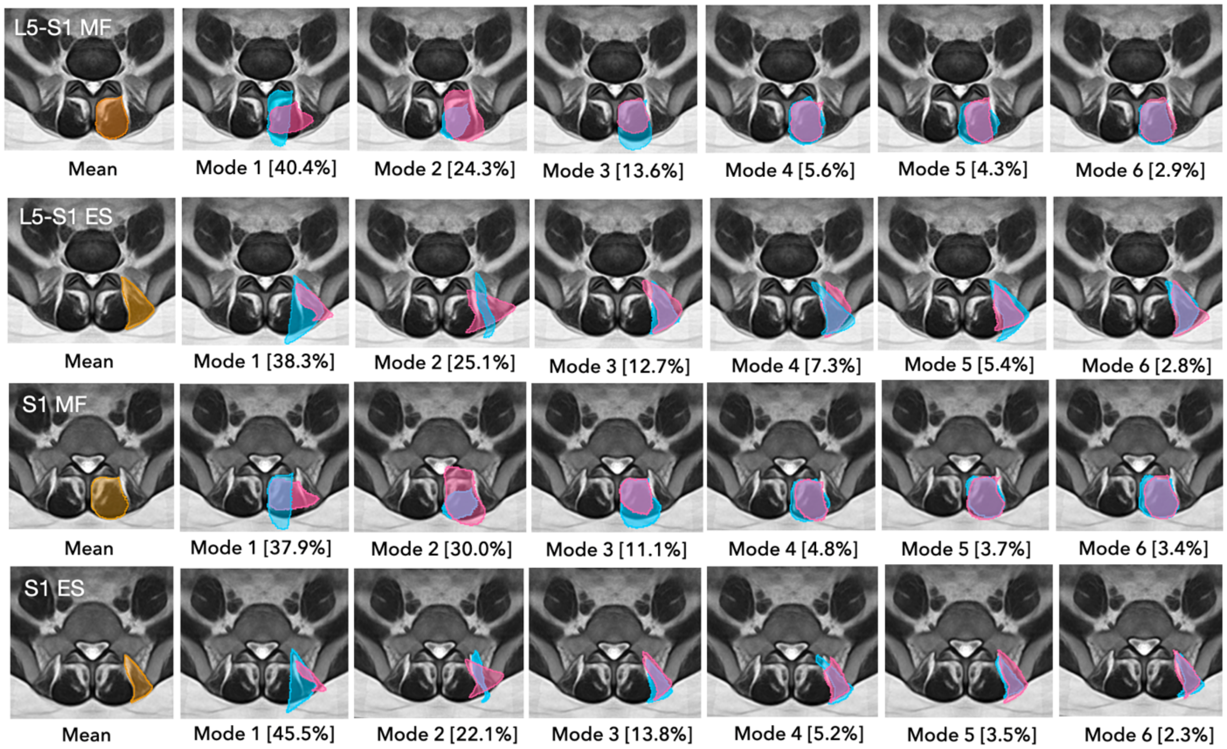

Figure S1. Shape modes for the multifidus (MF) and erector spinae (ES) at the L5-S1 and S1 levels. Here, the top 6 modes that account for ~90% of the shape variance for each muscle group at each level are shown. For each mode, 3-fold standard deviations from the mean shape are depicted for both directions of shape variants in blue and pink, and the percentages of shape variance explained are included in [ ] for all modes.

3. Correlation studies with shape variants

The top 6 modes that account for ~90% of muscle shape variations for each muscle group at each of the L5-S1 and S1 levels were correlated with sex, age, and the factor of affected vs. unaffected using Spearman partial correlation. When the shape mode was correlated with one factor, the effects of the other factors were controlled. The partial correlation results for all shape modes are included in Table S2.

	Mode	Affected vs. unaffected	Sex	Age
L5-S1 - MF	1	*| ρ |=0.148, p=0.326*	***| ρ |=0.323, p=0.029****	***| ρ |=0.520, p=0.0002****
	2	*| ρ |=0.132, p=0.380*	*| ρ |=0.009, p=0.954*	*| ρ |=0.039, p=0.796*
	3	*| ρ |=0.101, p=0.505*	*| ρ |=0.176, p=0.243*	*| ρ |=0.009, p=0.955*
	4	*| ρ |=0.089, p=0.556*	*| ρ |=0.269, p=0.070*	*| ρ |=0.275, p=0.065*
	5	*| ρ |=0.121, p=0.424*	*| ρ |=0.014, p=0.927*	*| ρ |=0.084, p=0.581*
	6	*| ρ |=0.122, p=0.421*	***| ρ |=0.514, p=0.0003****	***| ρ |=0.527, p=0.0002****
L5-S1 - ES	1	*| ρ |=0.084, p=0.581*	***| ρ |=0.331, p=0.025****	***| ρ |=0.439, p=0.002****
	2	*| ρ |=0.146, p=0.332*	*| ρ |=0.162, p=0.282*	*| ρ |=0.006, p=0.967*
	3	*| ρ |=0.105, p=0.489*	*| ρ |=0.015, p=0.923*	***| ρ |=0.308, p=0.037****
	4	*| ρ |=0.115, p=0.448*	*| ρ |=0.277, p=0.062*	*| ρ |=0.256, p=0.086*
	5	*| ρ |=0.144, p=0.339*	***| ρ |=0.438, p=0.002****	*| ρ |=0.216, p=0.149*
	6	*| ρ |=0.003, p=0.984*	*| ρ |=0.224, p=0.134*	*| ρ |=0.027, p=0.859*
S1- MF	1	*| ρ |=0.101, p=0.526*	***| ρ |=0.349, p=0.024****	***| ρ |=0.336, p=0.030****
	2	*| ρ |=0.045, p=0.777*	*| ρ |=0.240, p=0.125*	*| ρ |=0.112, p=0.480*
	3	*| ρ |=0.069, p=0.665*	*| ρ |=0.156, p=0.325*	*| ρ |=0.002, p=0.989*
	4	***| ρ |=0.332, p=0.032****	*| ρ |=0.083, p=0.604*	*| ρ |=0.118, p=0.458*
	5	*| ρ |=0.179, p=0.257*	***| ρ |=0.540, p=0.0002****	***| ρ |=0.311, p=0.045****
	6	***| ρ |=0.397, p=0.009****	*| ρ |=0.025, p=0.877*	*| ρ |=0.245, p=0.118*
S1- ES	1	*| ρ |=0.119, p=0.454*	***| ρ |=0.360, p=0.019****	*| ρ |=0.262, p=0.094*
	2	*| ρ |=0.069, p=0.664*	***| ρ |=0.335, p=0.030****	*| ρ |=0.047, p=0.766*
	3	*| ρ |=0.051, p=0.749*	*| ρ |=0.140, p=0.376*	*| ρ |=0.124, p=0.434*
	4	*| ρ |=0.130, p=0.413*	***| ρ |=0.597, p=0.00003****	***| ρ |=0.330, p=0.033****
	5	*| ρ |=0.120, p=0.450*	*| ρ |=0.291, p=0.062*	*| ρ |=0.051, p=0.750*
	6	*| ρ |=0.111, p=0.483*	*| ρ |=0.032, p=0.839*	*| ρ |=0.043, p=0.786*

Table S2. Partial correlations between top 6 modes (shape variations) and the designated factor while controlling for the rest. The absolute values of partial correlation | ρ | and the corresponding p-values are shown in the chart for each mode. Significant modes of each muscle group with respect to different factors ($p<0.05$) are marked with "*" and in bold.
